# Early Trends in Cystatin C and Outcomes in Patients with Cirrhosis and Acute Kidney Injury

**DOI:** 10.1155/2014/708585

**Published:** 2014-03-18

**Authors:** Justin M. Belcher, Arun J. Sanyal, Guadalupe Garcia-Tsao, Naheed Ansari, Steven G. Coca, Michael G. Shlipak, Chirag R. Parikh

**Affiliations:** ^1^Program of Applied Translational Research, Yale University School of Medicine, New Haven, CT 06510, USA; ^2^Section of Nephrology, Yale University School of Medicine, New Haven, CT 06520, USA; ^3^Clinical Epidemiology Research Center, VAMC, West Haven, CT 06516, USA; ^4^Division of Gastroenterology, Hepatology and Nutrition, Department of Internal Medicine, Virginia Commonwealth University School of Medicine, Richmond, VA 23298, USA; ^5^Section of Digestive Diseases, Yale University School of Medicine, New Haven, CT, USA; ^6^VA-Connecticut Healthcare System, West Haven, CT 06520, USA; ^7^Division of Nephrology, Department of Internal Medicine, Jacobi Medical Center, South Bronx, NY 10461, USA; ^8^Division of General Internal Medicine, San Francisco VA Medical Center, University of California, San Francisco, CA 94121, USA

## Abstract

*Background*. Acute kidney injury (AKI) is a common and severe complication in patients with cirrhosis. Progression of AKI to a higher stage associates with increased mortality. Intervening early in AKI when renal dysfunction is worsening may improve outcomes. However, serum creatinine correlates poorly with glomerular filtration in patients with cirrhosis and fluctuations may mask progression early in the course of AKI. Cystatin C, a low-molecular-weight cysteine proteinase inhibitor, is a potentially more accurate marker of glomerular filtration. *Methods*. We conducted a prospective multicenter study in patients with cirrhosis comparing changes in cystatin and creatinine immediately following onset of AKI as predictors of a composite endpoint of dialysis or mortality. *Results*. Of 106 patients, 37 (35%) met the endpoint. Cystatin demonstrated less variability between samples than creatinine. Patients were stratified into four groups reflecting changes in creatinine and cystatin: both unchanged or decreased 38 (36%) (Scr−/CysC−); only cystatin increased 25 (24%) (Scr−/CysC+); only creatinine increased 15 (14%) (Scr+/CysC−); and both increased 28 (26%) (Scr+/CysC+). With Scr−/CysC− as the reference, in both instances where cystatin rose, Scr−/CysC+ and Scr+/CysC+, the primary outcome was significantly more frequent in multivariate analysis, *P* = 0.02 and 0.03, respectively. However, when only creatinine rose, outcomes were similar to the reference group. *Conclusions*. Changes in cystatin levels early in AKI are more closely associated with eventual dialysis or mortality than creatinine and may allow more rapid identification of patients at risk for adverse outcomes.

## 1. Introduction

Acute kidney injury (AKI) is a common complication in patients with cirrhosis and associates with higher mortality in proportion to progressive AKI severity [1, 2]. However, the most common indicator of renal function, serum creatinine, may be an unreliable surrogate for glomerular filtration rate (GFR) due to the impact of nonrenal determinants such as sex, race, age, body composition, and medications. In the setting of an acute drop in GFR, creatinine is insensitive to small decrements in function, and its rise can lag actual kidney injury by several days. These shortcomings of creatinine are magnified in patients with cirrhosis, as they have an enlarged volume of fluid distribution and decreased creatinine production secondary to muscle atrophy and liver dysfunction, further dissociating creatinine from GFR [3]. The accuracy of creatinine in reflecting GFR declines with worsening stages of cirrhosis [4] and can be further compromised by elevated bilirubin interfering with creatinine assays [5]. We have previously shown that progression of AKI associates with mortality [6]. However, progression of AKI to a higher creatinine defined stage may be delayed in the setting of cirrhosis due to early fluctuation in creatinine levels unrelated to renal function and potentially beneficial treatments may resultantly be deferred. A more accurate means of rapidly and accurately detecting changes in renal function early in the course of AKI that associate with outcomes may allow for more prompt initiation of therapy and improved outcomes.

Cystatin C is a low-molecular-weight cysteine proteinase inhibitor synthesized at a constant rate by all nucleated cells. Cystatin C is freely filtered by the glomerulus, nearly completely reabsorbed and catabolized by the proximal tubule, and does not undergo secretion. Cystatin C levels are less influenced by nonrenal factors than creatinine and it has thus been proposed as a superior marker of glomerular filtration. In AKI, cystatin rises more rapidly than creatinine in some settings and has been shown to associate more strongly with outcomes. Cystatin performs better than creatinine in early detection of AKI in the emergency room [7], intensive care unit (ICU) [8, 9], and following pediatric cardiac surgery [10]. Cystatin associates with duration of AKI [11], need for renal replacement therapy [8, 12], and short and long term mortality in AKI [12, 13]. Patients who experience increases in both cystatin C and creatinine experience worse outcomes than those with an increase in either marker alone [14, 15]. In patients with cirrhosis, cystatin C has been shown to more accurately correlate with measured GFR than creatinine or creatinine based estimation equations [16]. Cystatin C is also more sensitive than creatinine in cirrhotics for detecting mild decreases in baseline GFR [17, 18] and superior in predicting AKI or 3-month mortality [19]. Despite these attributes, cystatin C has been challenging to study in patients with cirrhosis and AKI due to the typical lack of a documented baseline value. The absence of a baseline renders cystatin ineffectual in practice for diagnosing AKI prior to creatinine as there is no value to compare to for assessment of absolute or relative changes. However, due to its lesser dependence on nonrenal determinants, small changes in cystatin levels early in the course of AKI may be more reflective of true trends in renal function than those of creatinine, which might continue to oscillate for several days before displaying a clear trend towards renal worsening or recovery. An alternative study design therefore is comparing trends in cystatin C and creatinine levels immediately following the onset of clinical apparent AKI to evaluate the relative utility of early fluctuations in each marker in predicting outcomes following AKI. We conducted a prospective multicenter study in patients with cirrhosis comparing changes in cystatin C and creatinine immediately following onset of AKI as predictors of dialysis and mortality during this early time period.

## 2. Subjects and Methods

### 2.1. Study Design

The details of the cohort of patients with cirrhosis and AKI and study design have been described previously [6]. This prospective, multicenter observational cohort study was conducted between 2009 and 2011 at four tertiary care academic centers in the USA. Eligible patients were admitted with AKI (see “Section 2.3”) or developed it during the course of hospitalization. Inclusion criteria included a known diagnosis of cirrhosis (see “Section 2.3.1”), age ≥ 18 years, and availability of documented serum creatinine within 1 year prior to AKI. Exclusion criteria included prior kidney or liver transplant, advanced chronic kidney disease (CKD) (baseline creatinine >4.0 mg/dL), acute or chronic renal replacement therapy at enrollment, estimated life expectancy <3 days, confirmed pregnancy, and other known causes of renal insufficiency such as glomerulonephritis or urinary obstruction. Informed consent was obtained from all participants or, if patients were unable to provide consent, from designated surrogates. All consecutive eligible patients identified during screening were approached for enrollment and all participants were enrolled within 5 days of meeting AKI criteria. The study was approved by the institutional review board at each institution.

### 2.2. Sample Collection and Biomarker Measurement

A fresh 10 mL blood sample was collected daily for three days following the onset of AKI. Samples were immediately refrigerated and then centrifuged at 5000 ×g for 10 minutes at −4°C. Aliquots of 1 mL of supernatant were subsequently stored within 6 hours of collection in cryovials at −80°C for cystatin C measurement. No additives or protease inhibitors were utilized. Measurement was performed on subsequently thawed aliquots without undergoing any additional freeze-thaw cycles. Cystatin C was measured using a BN II nephelometer (Siemens AG, http://www.siemens.com/), which has an approximate coefficient of variation of 2% [20]. Creatinine was measured from samples collected as part of routine clinical care via the modified Jaffe method. Laboratory measurements were performed by personnel blinded to patient information.

### 2.3. Definitions

#### 2.3.1. Independent Variables

*Cirrhosis.* Patients who were eligible carried an existing documented diagnosis of cirrhosis based on liver biopsy, when available, or a combination of clinical, biochemical, ultrasonographic, and endoscopic findings.

*AKI.* The acute kidney injury network (AKIN) criteria were applied for diagnosis of AKI as recommended by a working group composed of members of the International Ascites Club (IAC) and the Acute Dialysis Quality Initiative (ADQI) [21]. AKIN quantifies the severity of AKI based on degree of increase in serum creatinine relative to baseline and is defined as follows: stage 1, increase in creatinine by 0.3 mg/dL or 50%; stage 2, 2- to 3-fold increase; stage 3, >3-fold increase, or creatinine >4.0 mg/dL after a rise of at least 0.5 mg/dL or acute dialysis requirement. As urine collection and output documentation can be inconsistent, only the serum creatinine component of the AKIN criteria was utilized. 

*Baseline Serum Creatinine*. Baseline serum creatinine was defined as the most recent stable measurement within a year prior to admission for the index hospitalization. When possible, outpatient measurements were utilized though values were also used from previous admissions not complicated by AKI. In rare cases, patients without an outpatient measurement were included in the analytic cohort if, prior to onset of AKI, they manifested at least 5 initial days from admission of stable values within the normal creatinine range. In these instances, the creatinine at admission was considered the baseline. 

*Other Variables*. When calculating between-sample percent change in creatinine and cystatin C, the first and last available samples were utilized. GFR was estimated via the CKD-EPI equation using the baseline creatinine value [22]. CKD was defined by as GFR < 60 mL/min. MELD and Child-Pugh scores were calculated on the day of first sample collection. 

*Outcomes*. Our primary outcome was a composite of dialysis and in-hospital mortality during the index hospitalization.

### 2.4. Statistics

Categorical variables were expressed as proportions and compared using Chi-square and Fisher's exact test, as appropriate. Normally or near normally distributed variables were reported as means with standard deviations (SD) and compared by Student's* t*-test. Nonnormally distributed continuous variables were reported as medians with interquartile ranges (IQR) and compared by Wilcoxon rank sum test. Normality was assessed using the Kolmogorov-Smirnov test. Correlation between the percentage change between samples of creatinine and cystatin C was assessed via Pearson's test.

Patients were categorized into four groups based on trends between the first and last sample of two filtration biomarkers, serum creatinine, and serum cystatin C. The groups were when (1) both biomarkers fell or were unchanged (2) only serum creatinine exhibited any increase (3) only serum cystatin C increased and (4) both increased. Only serum creatinine exhibited any increase, only serum cystatin C increased, and both increased. As our intent was to compare the association between small, early changes in filtration markers with outcomes, no threshold was utilized as to what constituted an increase. With the group with both biomarkers unchanged or falling as the reference, we determined crude and adjusted relative risks of each other group for our composite primary outcome with multivariate modified Poisson regression using SAS PROC GENMOD. Adjustment was made for critical demographics variables associated with filtration markers including race, age, and sex. Goodness-of-fit was verified with the Hosmer-Lemeshow test. A 2-sided *P* < 0.05 was considered significant for all analysis. Statistical analysis was performed using SAS, version 9.2 (SAS Institute, Cary, NC).

## 3. Results

A total of 192 patients were enrolled in our cohort with cirrhosis and AKI. Of these, 106 had at least 2 blood samples collected and were included in this study. Samples were not collected in the remaining 86 patients either due to failure to consent to blood collection or initiation of dialysis prior to obtaining consent. Baseline demographic, clinical, and laboratory characteristics for the entirety of study participants and the four groups designated by trends in creatinine and cystatin C are shown in Table 1. There were no significant differences in any demographic variables or in those relating to the patients' liver disease between those patients who did and did not have serum samples collected. The mean patient age was 56.3 and 66% were male. Thirty-seven (35%) patients met the primary composite endpoint during their hospitalization. Of these, 28 patients died and 22 required dialysis, with 13 of these experiencing both dialysis and death. On sensitivity analysis, there was no difference in death, 28/106 (26%) versus 22/86 (26%), or the composite of death or dialysis, 37/106 (35%) versus 30/86 (35%), between those patients with and without blood samples obtained. The majority of patients had advanced cirrhosis evidenced by previously suffered complications including ascites, 76%, hepatic encephalopathy, 63%, variceal bleeding, 23%, and SBP, 12%. Reasons for admission were similar between the four groups. The median Child-Pugh score was 10 and MELD 26.4 at the time of enrollment. There was no difference in Child-Pugh and MELD scores across groups nor were serum sodium levels or the presence of hyponatremia at enrollment significantly different.

### 3.1. Biomarkers and Prognosis

**Figure 1:**
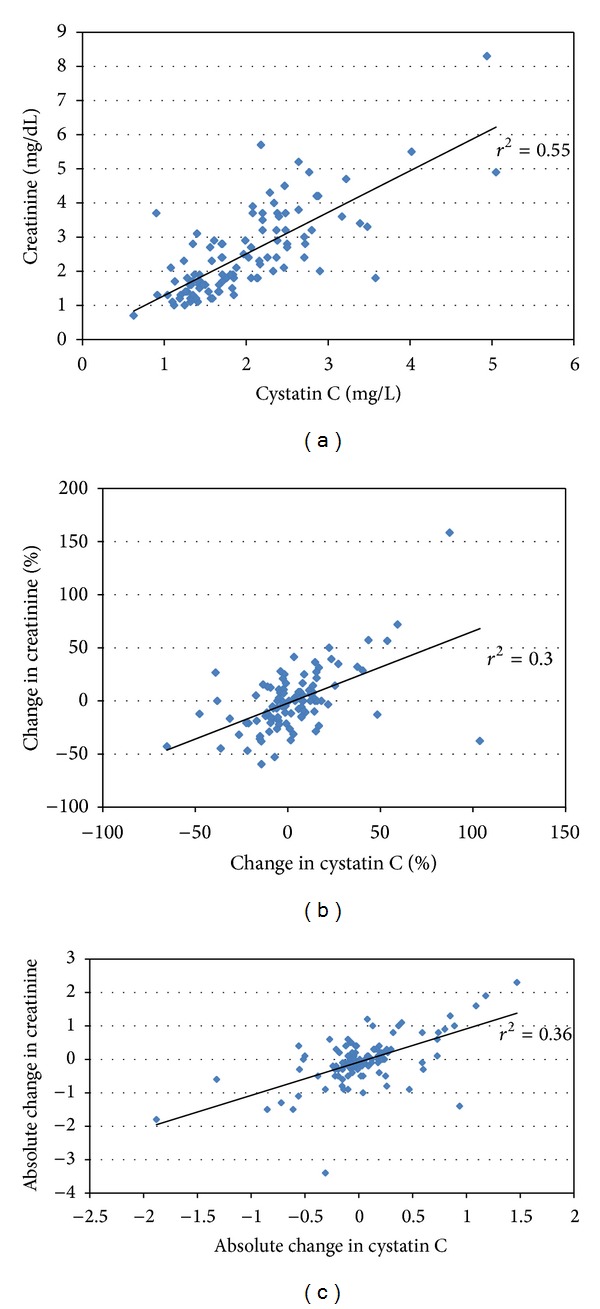
Correlation between creatinine and cystatin. (a) Correlation between creatinine and cystatin C values from first sample collection. (b) Correlation between relative changes in creatinine and cystatin C values from first to last sample collection. (c) Correlation between absolute changes in creatinine and cystatin C values from first to last sample collection.

Three blood samples were collected in 77 (73%) patients, and two were collected in the remainder, 29 (27%). The first sample was collected at a median of 2 (IQR 2–4) days after first meeting AKIN criteria. While creatinine and cystatin C levels from the first sample were moderately correlated, *r* ^2^ = 0.55, the relative changes in creatinine and cystatin C values between the first and last sample were less, so, *r* ^2^ = 0.3, *P* < 0.0001. Correlations between creatinine and cystatin C levels in the initial samples and between relative and absolute changes in each filtration marker between samples are shown in Figures 1(a), 1(b), and 1(c), respectively. Cystatin C exhibited less variability between samples than seen with creatinine with the interquartile range for percent change in creatinine ranging from −17 to +11% compared with cystatin C ranging from −9 to +12%. A change of <10% was observed in 35/106 (33%) patients by creatinine and 53/106 (50%) patients based on cystatin C (*P* = 0.018). The median change in cystatin C values differed significantly between those patients with the primary outcome, +6% (95% CI −2 to +14%), and those without, −3% (−9 to +9%), *P* = 0.03. The difference in changes in creatinine for those with and without the primary outcome trended in the same direction but did not reach statistical significance, 0% (−12 to +17%) versus −5% (−21 to +8%), *P* = 0.07. Patients experiencing an increase in cystatin C levels between samples were significantly more likely to meet the primary endpoint, 47%, than those without such an increase, 23%, *P* = 0.008. However, there was no significant difference in the incidence of dialysis or mortality among those whose creatinine increased, 40%, than among those where it did not, 32%, *P* = 0.41 (Table 2). Neither the cystatin C nor creatinine values from the first sample collected showed any association with the primary outcome.

Patients were stratified into four mutually exclusive groups based on changes in creatinine and cystatin C: both unchanged or decreased 38 (36%) (Scr−/CysC−); only cystatin C increased 25 (24%) (Scr−/CysC+); only creatinine increased 15 (14%) (Scr+/CysC−); and both creatinine and cystatin C increased 28 (26%) (Scr+/CysC+). The incidence of dialysis or death for each group is shown in Table 3. Taking the Scr−/CysC− group as the reference, in both instances where cystatin C rose, Scr−/CysC+ and Scr+/CysC+, the occurrence of the primary outcome was significantly higher, *P* = 0.02 and 0.03, respectively. However, in the group where only creatinine rose, outcomes were similar to the reference group. Both the Scr−/CysC+ and Scr+/CysC+ but not Scr+/CysC− groups were associated with a significantly increased relative risk for the primary outcome in unadjusted analysis as well as after adjustment for age, race, and sex.

## 4. Discussion

AKI in patients with cirrhosis is often severe and associated with significant mortality risk. Potentially efficacious therapies exist but must be appropriately applied to patients at highest risk for adverse outcomes [23]. We have demonstrated that progression to a more advanced stage of AKI is independently associated with mortality but the likelihood of progression can be difficult to predict early in the course of AKI. Creatinine levels are dependent on multiple demographic and clinical factors beyond renal function and thus may be susceptible to short term fluctuations early in the course of AKI unrelated to changing GFR [3, 24]. Cirrhosis potentiates these shortcomings of creatinine due to associated low protein intake, reduced muscle mass, defective creatinine production, and frequent large fluid shifts. In patients with cirrhosis, creatinine based estimation of GFR is within 50% of measured values in only 9% of patients [25]. Cystatin C has been proposed as a biomarker of glomerular filtration less susceptible to extrarenal variation. In patients with cirrhosis, GFR estimates are less biased and more precise with cystatin C than creatinine [25, 26]. Cystatin levels, but not creatinine, are associated in cirrhotic patients with development of AKI and mortality over a 3–6-month period [19] and the onset of hepatorenal syndrome and mortality at one year [27]. The purpose of this study was to compare the association of changes in cystatin C and creatinine early in the course of AKI in patients with cirrhosis with a composite outcome of dialysis or death.

In our study, changes in cystatin C, but not creatinine, over the period of sample collection differed significantly for those with and without the primary outcome. Participants experiencing a rise in cystatin C alone (Scr−/CysC+) between samples progressed to the need for dialysis or death at the same rate as those with a rise in both biomarkers of filtration (Scr+/CysC+). However, those with a rise in creatinine alone (Scr+/CysC−) experienced the primary outcome with no greater frequency than those in whom both biomarkers fell (Scr−/CysC−). Relative to the group in which both markers fell, both groups with rising cystatin were independently associated with the primary outcome. The lack of association between rising creatinine and our primary endpoint stands in contrast to our previous demonstration of a strong association between progression of AKI to a higher creatinine defined stage and mortality [6]. This discrepancy is again evidence of the poor sensitivity of creatinine for detecting acute falls in renal filtration function. Given its extrarenal influences and the extent to which changes in levels lag falling GFR, creatinine rising over the entire duration of an AKI episode sufficient to qualify for a higher AKI stage is indeed specific for a significant fall in renal function and resultantly associates with poor outcomes. Over the short term, however, early in the course of AKI, creatinine changes need not reflect trends in renal function and thus show poor association with outcomes when not coupled with similar changes in cystatin C levels.

Cystatin C strongly associates with outcomes in multiple settings of AKI including ICU^9^, emergency room [7], and transplant [28]. Intriguingly, changes in cystatin C may be more specific to outcomes than creatinine. Kwon et al. studied 274 ICU patients, of whom 84 (30.7%) developed AKI [29]. The mortality in patients with acute elevation in cystatin C but without creatinine based AKI (28.6%) was similar to patients with AKIN stage 1 AKI (33.3%) and far outstripped that of patients with no elevations in either biomarker (5.7%). This finding mirrors the results of our study with poor outcomes in patients with Scr−/Cys+. The apparent prognostic advantage of cystatin C may be due to its ability to more accurately reflect early/small changes in GFR due to fewer nonrenal influences. Early in AKI, before GFR has undergone a truly dramatic fall, creatinine may be subject to greater fluctuations than cystatin C, fluctuations untethered from changes in GFR. In our study, creatinine and cystatin C levels exhibited good correlation at time of first sample collection, *r* ^2^ = 0.55. However, the correlation between changes in these markers over the course of sample collection was significantly lower, *r* ^2^ = 0.3. Changes in cystatin C levels during the period of sample collection were more tightly bunched than those of creatinine. Cystatin C demonstrated less variability with a smaller interquartile range for changes and a significantly higher number of patients with a change of <10%.

In addition to being more specific for early changes in GFR than creatinine, cystatin C may also be more sensitive. The superiority of cystatin C over creatinine for detecting early acute changes in renal function has been noted in multiple settings. Herget-Rosenthal et al. performed daily serum collections on 85 ICU patients deemed high risk of developing AKI [8]. In the 44 (52%) patients who developed AKI as defined by the risk, injury, failure, loss, and end-stage (RIFLE) criteria, cystatin C levels detected AKI (defined by a 50% increase from baseline) 1.5 ± 0.6 days earlier than serum creatinine. Similar results have been noted in the ICU [8, 9], following iodinated contrast [30] and postoperatively in pediatric [10], though not adult [15], cardiac surgery.

Our study has several significant strengths. Data were collected prospectively for what is, in this challenging study population, a large cohort of patients. Unlike many studies of cirrhosis and AKI, ours was multicenter and contained patients from both general medical floors and the ICU, enhancing its generalizability. However, the study is not without limitations. Cystatin C can be influenced by several nonrenal factors including steroids and thyroid function. While we do not have data for these variables, it is reassuring that none of the baseline and demographic variables in Table 1 predicted which of the four groups patients would assort into. This is especially true for cirrhosis etiology, where the potential use of steroids to treat acute hepatitis in alcohol related cirrhosis did not dictate the pattern of changes in cystatin C. However, we cannot definitively rule out that changes in cystatin may be reflecting some other physiologic processes in addition to renal functions that may be contributing to the primary outcome. We did not have data on baseline cystatin C levels and patients were enrolled based on creatinine defined AKI. This raises a concern that the results could be biased for patients whose creatinine fell due to regression to the mean. However, the lack of association between enrollment cystatin and creatinine values and the primary outcome assuages this concern.

In conclusion, changes in serum cystatin C early in the course of AKI in patients with cirrhosis associate more strongly with the need for dialysis and mortality than do changes in serum creatinine. Prospective trials indexing interventions to changes in cystatin are required to determine if routine monitoring of cystatin C in patients with cirrhosis and AKI may lead to improved outcomes.

## Figures and Tables

**Table 1:** Baseline demographic, clinical, and laboratory values.

	Total	Scr−/CysC−	Scr−/CysC+	Scr+/CysC−	Scr+/CysC+	P
	*N* = 106	*N* = 38	*N* = 25	*N* = 15	*N* = 28	
Age in years, mean ± SD	56.3 ± 8.9	54.6 ± 9.8	57.1 ± 10.2	58.1	56.9	0.52
Male sex, *n* (%)	70 (66)	26 (68)	17 (68)	8 (53)	19 (68)	0.74
BMI, median (IQR)	30.6 (25.7–36)	32.2 (26.3–36.8)	29.2 (25.5–32.6)	33.2 (25.7–21.3)	31.5 (25–36.5)	0.41
Race, *n* (%)						
White	76 (72)	30 (79)	17 (68)	12 (80)	17 (61)	0.34
Black	16 (15)	4 (11)	4 (16)	2 (13)	6 (21)	0.67
Hispanic	12 (11)	4 (11)	3 (12)	1 (7)	4 (14)	0.90
Diabetes, *n* (%)	24 (23)	12 (32)	2 (8)	3 (20)	7 (25)	0.16
Active cancer, *n* (%)	13 (12)	5 (13)	2 (8)	2 (13)	4 (14)	0.92
Baseline creatinine mg/dL, median (IQR)	1.02 (0.8–1.3)	1 (0.8–1.2)	0.97 (0.8–1.2)	1.1 (0.9–1.43)	1.18 (0.8–1.56)	0.12
CKD^a^	34 (32)	9 (24)	8 (32)	6 (40)	11 (39)	0.51
Cirrhosis etiology, *n* (%)						
Alcohol	32 (30)	11 (29)	12 (38)	4 (27)	5 (18)	0.13
Alcohol and HCV	27 (25)	13 (34)	3 (12)	1 (7)	10 (36)	0.04
HCV	19 (18)	6 (16)	3 (12)	2 (13)	8 (29)	0.45
NASH	10 (9)	2 (5)	2 (8)	3 (20)	3 (11)	0.40
Cryptogenic	4 (4)	1 (3)	1 (4)	2 (13)	0 (0)	0.16
Autoimmune	7 (7)	3 (8)	2 (8)	2 (13)	0 (0)	0.28
Other	8 (8)	3 (8)	2 (8)	2 (13)	1 (4)	0.65
Previous complications of cirrhosis, *n* (%)						
Ascites	81 (76)	27 (71)	21 (84)	11 (73)	22 (79)	0.67
Hepatic encephalopathy	67 (63)	22 (58)	15 (60)	11 (73)	19 (68)	0.68
Variceal bleed	24 (23)	12 (32)	5 (20)	3 (20)	4 (14)	0.42
SBP	12 (12)	3 (8)	5 (20)	2 (13)	4 (14)	0.55
Reason for admission, *n* (%)						
Hepatic encephalopathy	26 (25)	10 (26)	4 (16)	5 (33)	7 (25)	0.63
Refractory ascites/edema	16 (15)	6 (16)	4 (16)	3 (20)	3 (11)	0.86
AKI	12 (11)	3 (8)	2 (8)	2 (13)	5 (18)	0.60
GI bleed	8 (8)	3 (8)	1 (4)	0 (0)	4 (14)	0.44
Abdominal pain	7 (7)	4 (11)	1 (4)	1 (7)	1 (4)	0.78
Jaundice	5 (5)	3 (8)	1 (4)	1 (7)	0 (0)	0.50
Transplant work-up	6 (6)	1 (3)	2 (8)	1 (7)	2 (7)	0.70
SBP	4 (4)	0 (0)	2 (8)	0 (0)	2 (7)	0.20
Infection other than SBP	4 (4)	2 (5)	1 (4)	1 (7)	0 (0)	0.57
Other	20 (19)	7 (18)	7 (28)	2 (13)	4 (14)	0.63
Child-Pugh Class^b^, *n* (%)						0.17
B	37 (35)	17 (45)	6 (24)	7 (47)	7 (25)	
C	69 (65)	21 (55)	19 (76)	8 (53)	21 (75)	
Child-Pugh score, median (IQR)	10 (9–12)	10 (9–12)	11 (10–12)	10 (8–12)	10 (10–12)	0.39
MELD score, mean ± SD	26.4 ± 9.5	25 ± 9	26.8 ± 9.8	23.8 ± 7.4	29.3 ± 10.5	0.20
Bilirubin, median (IQR)	4 (1.8–9.1)	3 (1.6–6.4)	6.1 (2.6–9.6)	3.8 (1.6–5.5)	5.5 (2–16.7)	0.31
INR, median (IQR)	1.7 (1.3–2.3)	1.5 (1.2–2.3)	1.8 (1.4–2.3)	1.6 (1.2–1.8)	1.9 (1.4–2.7)	0.08
Sodium, mean ± SD	133 ± 6	133 ± 6	132 ± 7	135 ± 7	133 ± 7	0.74
Hyponatremia at enrollment^c^, *n* (%)	34 (32)	12 (32)	10 (40)	5 (33)	7 (25)	0.71

^a^CKD defined as GFR < 60 mL/min calculated via CKD-EPI equation.

^b^Child-Pugh Class and MELD score at time of enrollment.

^c^Serum sodium <130 mEq/L.

SD: standard deviation; BMI: body mass index; IQR: interquartile range; CKD: chronic kidney disease; HCV: hepatitis C virus; NASH: nonalcoholic steatohepatitis; SBP: spontaneous bacterial peritonitis; MELD: model of end-stage liver disease; INR: international normalized ratio.

**Table 2:** Association between increasing filtration markers and the primary outcome.

	Death/dialysis, *N* (%)	Dialysis-free survival, *N* (%)	*P*
*Creatinine *			
Increase (*N* = 43)	17 (40)	26 (60)	0.41
No increase (*N* = 63)	20 (32)	43 (68)	
*Cystatin C *			
Increase (*N* = 53)	25 (47)	28 (53)	0.008
No increase (*N* = 53)	12 (23)	41 (77)	

**Table 3:** Independent association of trends in filtration markers and the primary outcome.

	Death or dialysis, *N* (%)	Death or dialysis	
		Unadjusted RR (95% CI)	Adjusted* RR (95% CI)
Scr−/CysC− (*N* = 38)	8 (21)	1.00	1.00
Scr−/CysC+ (*N* = 25)	12 (48)	2.28 (1.09–4.77)	2.27 (1.07–4.85)
Scr+/CysC− (*N* = 15)	4 (27)	1.27 (0.45–3.59)	1.32 (0.46–3.75)
Scr+/CysC+ (*N* = 28)	13 (46)	2.21 (1.06–4.59)	2.17 (1.03–4.61)

*Adjusted for race, age, and sex.

## References

[1] Arabi Y, Ahmed QAA, Haddad S, Aljumah A, Al-Shimemeri A (2004). Outcome predictors of cirrhosis patients admitted to the intensive care unit. *European Journal of Gastroenterology and Hepatology*.

[2] Cholongitas E, Calvaruso V, Senzolo M (2009). RIFLE classification as predictive factor of mortality in patients with cirrhosis admitted to intensive care unit. *Journal of Gastroenterology and Hepatology*.

[3] Francoz C, Glotz D, Moreau R, Durand F (2010). The evaluation of renal function and disease in patients with cirrhosis. *Journal of Hepatology*.

[4] Ustundag Y, Samsar U, Acikgoz S (2007). Analysis of glomerular filtration rate, serum cystatin C levels, and renal resistive index values in cirrhosis patients. *Clinical Chemistry and Laboratory Medicine*.

[5] Cholongitas E, Marelli L, Kerry A (2007). Different methods of creatinine measurement significantly affect MELD scores. *Liver Transplantation*.

[6] Belcher JM, Garcia-Tsao G, Sanyal A (2012). Association of AKI with mortality and complications in hospitalized patients with cirrhosis. *Hepatology*.

[7] Soto K, Coelho S, Rodrigues B (2010). Cystatin C as a marker of acute kidney injury in the emergency department. *Clinical Journal of the American Society of Nephrology*.

[8] Herget-Rosenthal S, Marggraf G, Hüsing J (2004). Early detection of acute renal failure by serum cystatin C. *Kidney International*.

[9] Nejat M, Pickering JW, Walker RJ, Endre ZH (2010). Rapid detection of acute kidney injury by plasma cystatin C in the intensive care unit. *Nephrology Dialysis Transplantation*.

[10] Zappitelli M, Krawczeski CD, Devarajan P (2011). Early postoperative serum cystatin C predicts severe acute kidney injury following pediatric cardiac surgery. *Kidney International*.

[11] Haase M, Bellomo R, Devarajan P (2009). Novel biomarkers early predict the severity of acute kidney injury after cardiac surgery in adults. *Annals of Thoracic Surgery*.

[12] Haase-Fielitz A, Bellomo R, Devarajan P (2009). Novel and conventional serum biomarkers predicting acute kidney injury in adult cardiac surgery—a prospective cohort study. *Critical Care Medicine*.

[13] Bell M, Granath F, Mrtensson J, Löfberg E, Ekbom A, Martling C-R (2009). Cystatin C is correlated with mortality in patients with and without acute kidney injury. *Nephrology Dialysis Transplantation*.

[14] Briguori C, Visconti G, Rivera NV (2010). Cystatin C and contrast-induced acute kidney injury. *Circulation*.

[15] Spahillari A, Parikh CR, Sint K (2012). Serum cystatin C-versus creatinine-based definitions of acute kidney injury following cardiac surgery: a prospective cohort study. *American Journal of Kidney Diseases*.

[16] Hoek FJ, Kemperman FAW, Krediet RT (2003). A comparison between cystatin C, plasma creatinine and the Cockcroft and Gault formula for the estimation of glomerular filtration rate. *Nephrology Dialysis Transplantation*.

[17] Gerbes AL, Gülberg V, Bilzer M, Vogeser M (2002). Evaluation of serum cystatin C concentration as a marker of renal function in patients with cirrhosis of the liver. *Gut*.

[18] Demirtaş S, Bozbaş A, Akbay A, Yavuz Y, Karaca L (2001). Diagnostic value of serum cystatin C for evaluation of hepatorenal syndrome. *Clinica Chimica Acta*.

[19] Chung MY, Jun DW, Sung SA (2010). Diagnostic value of cystatin C for predicting acute kidney injury in patients with liver cirrhosis. *The Korean Journal of Hepatology*.

[20] Erlandsen EJ, Randers E, Kristensen JH (1999). Evaluation of the dade behring N latex cystatin C assay on the dade behring nephelometer II system. *Scandinavian Journal of Clinical and Laboratory Investigation*.

[21] Wong F, Nadim MK, Kellum JA (2011). Working Party proposal for a revised classification system of renal dysfunction in patients with cirrhosis. *Gut*.

[22] Levey AS, Stevens LA, Schmid CH (2009). A new equation to estimate glomerular filtration rate. *Annals of Internal Medicine*.

[23] Dobre M, Demirjian S, Sehgal AR, Navaneethan SD (2011). Terlipressin in hepatorenal syndrome: a systematic review and meta-analysis. *International Urology and Nephrology*.

[24] Davenport A (2011). Difficulties in assessing renal function in patients with cirrhosis: potential impact on patient treatment. *Intensive Care Medicine*.

[25] Pöge U, Gerhardt T, Stoffel-Wagner B, Klehr HU, Sauerbruch T, Woitas RP (2006). Calculation of glomerular filtration rate based on Cystatin C in cirrhotic patients. *Nephrology Dialysis Transplantation*.

[26] Demirtaş S, Bozbaş A, Akbay A, Yavuz Y, Karaca L (2001). Diagnostic value of serum cystatin C for evaluation of hepatorenal syndrome. *Clinica Chimica Acta*.

[27] Ahn HS, Kim YS, Kim SG (2012). Cystatin C is a good predictor of hepatorenal syndrome and survival in patients with cirrhosis who have normal serum creatinine levels. *Hepatogastroenterology*.

[28] Hall IE, Doshi MD, Poggio ED, Parikh CR (2011). A comparison of alternative serum biomarkers with creatinine for predicting allograft function after kidney transplantation. *Transplantation*.

[29] Kwon SH, Hyun J, Jeon JS, Noh H, Han DC (2011). Subtle change of cystatin C, with or without acute kidney injury, associated with increased mortality in the intensive care unit. *Journal of Critical Care*.

[30] Bachorzewska-Gajewska H, Malyszko J, Sitniewska E (2008). NGAL (neutrophil gelatinase-associated lipocalin) and cystatin C: are they good predictors of contrast nephropathy after percutaneous coronary interventions in patients with stable angina and normal serum creatinine?. *International Journal of Cardiology*.

